# Cost-effectiveness of radiofrequency echographic multi-spectrometry for the diagnosis of osteoporosis in the United States

**DOI:** 10.1093/jbmrpl/ziae138

**Published:** 2024-11-06

**Authors:** Jean-Yves Reginster, Stuart L Silverman, Majed Alokail, Nasser Al-Daghri, Mickael Hiligsmann

**Affiliations:** Biochemistry Department, College of Science, King Saud University, Riyadh 11451, Kingdom of Saudi Arabia; Cedars-Sinai Medical Center, Los Angeles and the OMC Clinical Research Center, Beverly Hills, CA 90211, United States; Biochemistry Department, College of Science, King Saud University, Riyadh 11451, Kingdom of Saudi Arabia; Biochemistry Department, College of Science, King Saud University, Riyadh 11451, Kingdom of Saudi Arabia; Department of Health Services Research, CAPHRI Care and Public Health Research Institute, Maastricht University, Maastricht, 6229 GT, The Netherlands

**Keywords:** cost-effectiveness, diagnosis, economic, fracture prevention, osteoporosis, REMS

## Abstract

Radiofrequency echographic multi-spectrometry (REMS) is an innovative, non-ionizing diagnostic technique that has shown high accuracy and precision, making it a promising alternative to DXA for osteoporosis diagnosis in clinical settings. With economic considerations playing an increasingly crucial role in healthcare decisions, this study aims to evaluate the cost-effectiveness and economic impact of improved osteoporosis diagnosis using REMS followed by treatment in the United States. A microsimulation-based Markov model was constructed to estimate the cost per quality-adjusted life year (QALY) gained (in US$2022) for REMS followed by treatment vs no diagnosis and treatment in US women aged 50 yr and older with osteoporosis. Women were categorized as high risk (receiving alendronate monotherapy for 5 yr) or very high risk (receiving an 18-mo course of anabolic treatment, abaloparatide, followed by 5 yr of alendronate). The study evaluated 2 medication adherence scenarios: one assuming full adherence to treatment and the other reflecting real-world adherence. The results indicate that REMS followed by treatment is associated with improved health outcomes, including more QALYs and fewer fractures, and reduced fracture-related costs compared to no diagnosis and treatment. The incremental cost-effectiveness ratio of REMS was estimated at $33 891 and $49 198 per QALY gained, under the full adherence and real-world adherence scenarios, respectively. These values are below the US cost-effectiveness threshold of $100 000 per QALY. Moreover, a 5% increase in the diagnosis and treatment of women over 50 yr at high and very high risk of fractures using REMS is projected to save approximately 30 000 life yr, 43 500 QALYs, and prevent 100 000 fractures over a lifetime under real-world medication adherence. In conclusion, this study suggests that REMS is a cost-effective strategy for the diagnosis and management of osteoporosis in US women, offering substantial potential economic benefits and improved health outcomes.

## Graphical Abstract

**Graphical Abstract:**
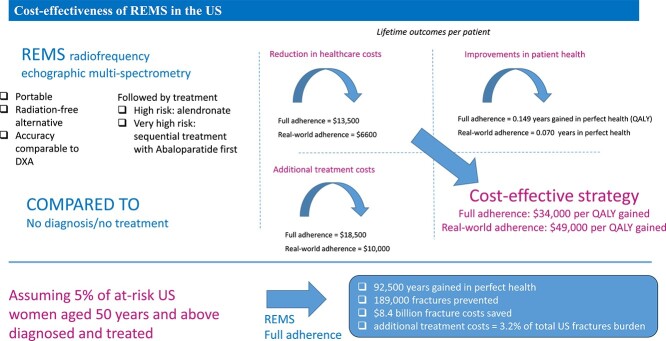

## Introduction

Osteoporotic-related fractures represent a substantial and escalating burden on patients, healthcare providers, policymakers, and society. It is estimated that 1 in 4 men and 1 in 2 women aged 50 yr and older will experience an osteoporotic fracture during their remaining lifetime.^1^ Fractures, particularly those of the hip or spine, are associated with increased morbidity and mortality and significantly impact quality of life. In 2019, the 27 countries of the European Union, plus the United Kingdom and Switzerland, experienced an estimated 4.3 million fragility fractures among individuals aged 50 and older, with a total economic burden estimated at €57 billion.^2^ Due to the anticipated aging and expansion of the population, the annual incidence of fractures in the United States is expected to climb from 1.9 million in 2018 to 3.2 million by 2040, a 68% increase. Correspondingly, the associated costs are predicted to rise from $57 billion to over $95 billion.^3^ Despite the availability of effective and safe medications, an estimated 71% of patients at high risk of fracture remain untreated,^2^ partly due to inadequate diagnostic practices. Timely diagnosis is therefore critical to mitigating the growing burden of osteoporotic fractures.

DXA is the most commonly used method for assessing BMD. However, emerging technologies that do not utilize radiation are garnering attention for the early diagnosis of compromised bone health and the fracture prevention. Radiofrequency echographic multi-spectrometry (REMS) is a novel, non-ionizing diagnostic approach that has recently been introduced.^4–6^ Clinical studies have demonstrated that REMS offers a high level of accuracy and precision comparable to DXA.^7^^,^^8^ Consequently, REMS shows promise for enhancing osteoporosis diagnosis in routine clinical practice as an alternative to DXA technique.^9^ An Italian study recently indicated that REMS is associated with lower direct healthcare costs compared to DXA.^10^ However, a comprehensive economic evaluation of REMS, in terms of costs per quality-adjusted life year (QALY) gained, has not yet been conducted. Such evaluations are essential for informing payers about the cost-effectiveness of REMS, as recommended by the ESCEO-IOF guideline for economic evaluations in osteoporosis.^11^

The aim of this study is to estimate the cost-effectiveness and economic impact (in terms of health outcomes and costs) of REMS for the diagnosis and treatment of osteoporosis in the United States. This analysis will provide crucial insights into the cost-effectiveness and potential economic benefits of implementing REMS in clinical practice.

## Materials and methods

The study followed the recommendations for conducting and reporting economic evaluations in osteoporosis as outlined by the ESCEO-IOF^11^ and the Consolidated Health Economic Evaluation Reporting Standards (CHEERS) 2022 statement.^12^ The completed CHEERS 2022 checklist, as well as the ESCEO-IOF checklists for reporting economic evaluations, and for the design and conduct of economic evaluations in osteoporosis are included in Appendix A.

The economic model and the majority of fracture and treatment data utilized in this study are derived from a recent evaluation of the cost-effectiveness of sequential treatment with abaloparatide (ABL) in the United States.^13^^,^^14^ The subsequent sections provide a description of the model, covering the target population and fracture risk, fracture costs and quality of life, treatment and diagnosis, and various analyses. Key model parameters are presented in Table 1 and described below. Previous publications also offer useful rationale for model components and assumptions.^13^^,^^14^

**Table 1:** Key model parameters.

**Parameter**	**Data**	
**Age distribution**	11% (50-54 y), 11.2% (55-59 y), 11.5% (60-64 y), 21.1% (65-69 y), 17.7% (70-74 y), 12.0% (75-79 y), 8.0% (80-84 y), 7.6% (85+ y)	
**Incidence (annual rate per 100) of fracture^20^**		
** Hip**	0.029 (50-54 y), 0.057 (55-59 y), 0.105 (60-64 y), 0.203 (65-69 y), 0.394 (70-74 y), 0.793 (75-79 y), 1.447 (80-84 y), 2.606 (85+ y)	
** Vertebral**	0.064 (50-54 y), 0.132 (55-59 y), 0.124 (60-64 y), 0.233 (65-69 y), 0.473 (70-74 y), 0.523 (75-79 y), 0.622 (80-84 y), 1.095 (85+ y)	
** NHNV**	0.820 (50-54 y), 1.340 (55-59 y), 1.597 (60-64 y), 1.722 (65-69 y), 2.106 (70-74 y), 2.722 (75-79 y), 3.256 (80-84 y), 3.923 (85+ y)	
**Increased relative risk due to osteoporosis**		
** Hip**	5.659 (50-59 y), 3.390 (60-69 y), 2.250 (70-79 y), 1.570 (80+)	
** Vertebral**	2.680 (50-59 y), 2.176 (60-69 y), 1.772 (70-79 y), 1.514 (80+)	
** NHNV**	2.250 (50-59 y), 1.902 (60-69 y), 1.610 (70-79 y), 1.416 (80+)	
**Increased relative risk of subsequent fracture after a fracture**		
** First fracture**	2.1 (0-6 M), 2.0 (7-12 M), 1.9 (13-18 M), 1.7 (19-24 M), 1.6 (25-36 M), 1.5 (37-48 M), 1.5 (49 M+)	
** Second and more fracture**	2.4 (0-6 M), 2.1 (7-12 M), 1.8 (13-18 M), 1.7 (19-24 M), 1.7 (25-36 M), 1.5 (37-48 M), 1.5 (49 M+)	
**Mortality excess^11^^,^^44^**		
** Hip (0-6 m/7-12 m/subs. y)**	4.54 (3.56-5.88)/1.76 (1.43-2.16)/1.78 (1.33-2.39)	
** Vertebral (0-6 m/7-12 m/subs. y)**	4.54 (3.56-5.88)/1.76 (1.43-2.16)/1.78 (1.33-2.39)	
** NHNV (0-12 m)**	1.38 (1.18-1.62)	
** % attributable to Fx**	25%	
**First-year cost of a subsequent fracture (estimated in US$2022) (adjusted from^28^)**		
** Hip**	119 613 (50-64 y), 75 658 (65+ y)	
** Vertebral**	60 459 (50-64 y), 35 006 (65+ y)	
** NHNV**	29 013 (50-64 y), 31 764 (65+ y)	
**Fracture costs (estimated in US$2022) for year 2 up to year 5 (adjusted from^32^)**		
** Hip**	Commercial: 10 804 (year 2), 7550 (year 3), 5947 (year 4), 3555 (year 5+) Medicare: 7654 (year 2), 5688 (year 3), 4052 (year 4), 2898 (year 5+)	
** Vertebral**	Commercial: 8196 (year 2), 4528 (year 3), 2566 (year 4), 1746 (year 5) Medicare: 5760 (year 2), 4094 (year 3), 2932 (year 4), 2170 (year 5)	
** NHNV**	Commercial: 1757 (year 2), 1097 (year 3), 642 (year 4), 377 (year 5) Medicare: 2340 (year 2), 2025 (year 3), 1335 (year 4), 1263 (year 5)	
**Health state utility values**		
**Baseline utility**	0.837 (50-59 y), 0.706 (60-69 y), 0.671 (70-79 y), 0.630 (80+ y)	
** RR after hip (1st y/subs. y)**	0.55 (0.53-0.57)/0.86 (0.84-0.89)	
** RR after vertebral (1st y/subs. y)**	0.68 (0.65-0.70)/0.85 (0.82-0.87)	
** RR after NHNV (1st y/subs. y)**	0.79 (0.65-0.93)/0.95 (0.81-1.09)	
	ABL	Generic ALN
**Effects on fracture (expressed as relative risk compared to placebo) of medications^33^^,^^34^**		
** Hip**	0.63 (0.41-0.98)	0.67 (0.48-0.96)
** Vertebral**	0.16 (0.06-0.42)	0.45 (0.31-0.65)
** NHNV**	0.42 (0.25-0.70)	0.81 (0.68-0.97)
**Drug cost (US$ per year)**	24 600	390
**REMS cost (US$)**	72.76	
**Persistence rate^36^**	59.1%	35.1% (17.5% from year 3)
**Number of osteoporosis patients eligible for treatment that could be diagnosed by REMS, assuming 5% of all patients at HR and VHR diagnosed and treated**		
**Very high risk patients**	33 302 (50-54 y), 34 020 (55-59 y), 34 996 (60-64 y), 64 108 (65-69 y), 53 840 (70-74 y), 36 353 (75-79 y), 24 218 (80-84 y), 23 043 (85+ y)	
** High risk patients**	35 080 (50-54 y), 35 836 (55-59 y), 36 864 (60-64 y), 67 530 (65-69 y), 56 714 (70-74 y), 38 294 (75-79 y), 25 511 (80-84 y), 24 273 (85+ y)	

Abbreviations: ABL, abaloparatide; ALN, alendronate; M, months; NHNV, non-hip non-vertebral; RR, relative reduction; subs., subsequent; y, years.

### Economic model

Consistent with previous studies and recommendations,^11^^,^^13^^,^^14^ we employed a Markov microsimulation model to track fracture events per patient and simulate health outcomes and healthcare costs over a lifetime, up to 100 yr.^11^^,^^15^ The model included 5 health states: no fracture, death, hip fracture, vertebral fracture, and non-hip non-vertebral (NHNV) fractures. Patients could experience multiple fractures at the same site or across different sites. A discount rate of 3% for both costs and health outcomes was applied, as recommended in the United States.^16^ The model was developed using TreeAge Pro 2023 R2.1 software (TreeAge Pro Inc., Williamston, MA).

### Target population and fracture risk

Analyses were conducted on US women aged 50 yr and older with osteoporosis, who represented the starting cohort of the model, categorized into high risk (HR) and very high risk (VHR) of fractures, further divided into 5-yr age groups. Of these women, 33.7% were aged between the ages of 50 and 64 yr. Based on 2017-2018 NHANES data,^17^ the prevalence of osteoporosis, affecting the femoral neck, lumbar spine, or both, was estimated at 13.1% for women aged 50-64 yr and 27.1% for those aged 65 and older. These rates were applied to the 2022 US population, derived from Census data, segmented by 5-yr age groups. Among women diagnosed with osteoporosis, 48.7% were classified as VHR and 51.3% as HR, according to Diffenderfer et al.^18^ who used one or more of the following characteristics to identify patients at VHR of fractures in a large claim database: a history of fracture while receiving osteoporosis therapy; multiple fractures during the observation period; fractures within the past 12 mo; fractures occurring while on medications with known adverse effects on bone (eg, long-term glucocorticoids); and/or a history of falls resulting in injury or a comorbidity associated with a HR of falls, fractures, or poor bone quality. In the model, women at VHR were assumed to have had a recent fracture in addition to densitometric osteoporosis, consistent with AACE/ACE criteria.^19^ Those at HR were assumed to have densitometric osteoporosis without any previous fractures.

Four potential components made up the model’s fracture risk: the fracture risk of the general women population, the increased risk of fracture linked to densitometric osteoporosis, the increased risk of fracture as a result of a recent fracture, and the potential reduction in fracture risk resulting from treatment. The incidences of hip and vertebral fractures in the US general population were derived from the study of Ettinger et al.,^20^ the same study used to develop the current US FRAX scores. Although the incidence rates are based on data from Ettinger et al.^20^ from more than a decade ago, recent studies, including Lewiecki et al.,^21^ show that fracture rates in the United Stateshave stabilized or increased since 2014, supporting the continued relevance of these data. These rates are detailed by 5-yr age groups and have been used in recent economic evaluations in the United States,^13^^,^^14^ making them suitable for our model. The increased risk due to osteoporosis (BMD T-score ≤ −2.5) was based on a previously validated method^22^ and the increased risk of fractures due to a previous fracture was based on a large Swedish study that reported the risk of a subsequent fracture according to time since fractures and number of fractures.^23^

Baseline mortality rates for age-stratified US women (estimated in 2020) were obtained from official estimates (National Vital Statistics System). Consistent with previous economic studies, increased mortality after hip, vertebral, and NHNV fractures were included in the model.^13^^,^^24^ The excess mortality of NHNV was derived from the study of Tran et al.^25^ Because excess mortality may also be attributable to comorbidities, only 25% of the excess mortality following fractures was assumed to be attributable to the fractures themselves.^26^^,^^27^

### Fracture costs and quality of life

Our analysis adopts a healthcare sector perspective and all healthcare costs were expressed in 2022 US dollars using the US Consumer Price Index for medical care where applicable. Incremental costs within 5 yr following hip, vertebral, and NHNV fractures were derived from Tran et al.,^28^ which estimated Medicare and commercial costs for fracture patients compared to controls. For vertebral and NHNV fractures, no additional costs were assumed beyond the initial 5-yr period. However, for hip fractures, the year 5 incremental cost from Tran et al.’s study^28^ was extrapolated over the patient’s lifetime due to long-term nursing home admissions and associated costs. In cases of multiple fractures, only the highest fracture cost was considered. Utility data were derived from the “Report of Nationally Representative Values for the Noninstitutionalized US Adult Population for Five Health-Related Quality-of-Life Scores” (using EQ-5D).^29^ The impact of fractures on utility was derived from the International Costs and Utilities Related to Osteoporotic Fractures Study (ICUROS), which is the largest study of its kind, assessing the quality of life of 3021 patients (86% women) with fractures across 11 countries.^30^

### Treatment and diagnosis

We compared REMS followed by treatment to a scenario with no diagnosis and no treatment. This comparator not only reflects real-world situations where diagnostic programs are lacking and most patients remain untreated, but also provides a clearer assessment of the incremental health and economic benefits offered by diagnostic interventions, which might be less evident when using more active comparators.

Based on clinical studies demonstrating that REMS achieves accuracy and precision comparable to DXA,^7^^,^^8^ REMS was assumed to accurately detect all patients diagnosed with osteoporosis in base case. Patients classified as HR received oral bisphosphonate treatment (specifically alendronate [ALN]) for 5 yr, while those classified as VHR underwent sequential therapy. This involved initiating treatment with an anabolic agent (ABL) for 18 mo, followed by 5 yr of ALN, as per the AACE/ACE guidelines.^17^ ABL was selected as a cost-effective anabolic agent in the United States.^13^ Sequential treatments for osteoporosis have been increasingly adopted in the United States, as they align with current clinical guidelines advocating for sequential treatment with an anabolic agent followed by an anti-resorptive in VHR patients. Recent studies suggest that this sequential therapy can enhance treatment effectiveness and outcomes, particularly for those at VHR of fractures.^31^

Similar treatment assumptions as previous studies in women with postmenopausal osteoporosis were applied for both sequential and monotherapy treatments.^24^^,^^32^ In the sequential strategy ABL followed by ALN, the fracture risk reduction with ABL over 43 mo was based on the ACTIVE/ACTIVExtend ITT trial data. ABL reduced the risk of vertebral fractures by 84% (RR 0.16; 95% CI 0.06-0.42) and non-hip, non-vertebral (NHNV) fractures by 58% (RR 0.42; 95% CI 0.25-0.70). For hip fractures, we conservatively assumed a 37% risk reduction with ABL (RR 0.63; 95% CI 0.41-0.98), extrapolated from NHNV fracture data.^33^ Upon starting ALN treatment, fracture risk reduction was assumed to follow similar proportions as in treatment-naive patients, using estimates from the National Institute for Health and Care Excellence (NICE) meta-analysis (TA464).^34^ ALN therefore reduced the risk of hip fractures by 33% (RR 0.67; 95% CI 0.48-0.96), vertebral fractures by 55% (RR 0.45; 95% CI 0.31-0.65), and NHNV fractures by 19% (RR 0.81; 95% CI 0.68-0.97). Consistent with ACTIVExtend findings, which suggest that the effects of a bone-forming agent like ABL persist when switching to an anti-resorptive drug like ALN, it was assumed that the effects of ABL remain constant during ALN treatment, with a conservative linear decrease over an additional year post-ALN discontinuation. The effects of ALN were assumed to decline linearly to zero over a similar period as the treatment duration, in line with previous economic studies.^11^

Drug prices were obtained from the wholesale acquisition cost listed in the Online Red Book (May 2022). The annualized costs for ABL and generic ALN were estimated at $24 600 and $182, respectively. Additional costs included one general physician visit ($118) every 6 mo during treatment. Base case costs for REMS were assumed to be comparable to Medicare reimbursement rates for DXA, estimating $72.76. Additional REMS assessments were scheduled every 2 yr for patients undergoing treatment. Adverse events associated with medications, such as hypercalcemia with ABL and gastrointestinal risks with ALN, were incorporated into the analysis following methodologies used in previous economic studies.^13^

Two different adherence scenarios were explored: complete medication adherence throughout the treatment period and real-world medication adherence that reflects current real-world cost-effectiveness. The real-world adherence scenario was derived from recent studies, adjusting treatment effects and costs based on medication persistence using formulas from Liu et al.^35^ Persistence data from Cheng et al.^36^ were used to assess how well patients stick to their osteoporosis medications. This study looked at 10 863 US women who started osteoporosis treatments, including anabolic treatment, teriparatide (TPTD), and oral bisphosphonates like ALN. The 12-mo persistence rate for TPTD, applied to ABL in the model, was 59.1%, matching findings from other studies. Persistence for ALN at 12 mo was 35.1%, similarly to another US study from Singer et al.^37^ A lower persistence rate of 17.5% was assumed for ALN from year 3 onwards.^37^

### Analyses

Based on 2000 000 individual simulations, the model estimated total healthcare costs, fracture incidences, life years, and QALYs for both the REMS followed by treatment and no REMS/treatment scenarios. The primary outcome assessed was the incremental cost-effectiveness ratio (ICER), which measures the additional costs required by the REMS/treatment strategy to gain one additional QALY. In the United States, the Institute for Clinical and Economic Review suggests that strategies with a cost per QALY gained lower than US$100000 are considered high value in healthcare.^38^

Multiple sensitivity analyses were conducted to assess the robustness of the results. In addition to the 2 medication adherence scenarios, 1-way sensitivity analyses were performed by varying one parameter input at a time across model parameters and structure. These included varying fracture incidence (±25%), fracture costs (±25%), effects of fractures on utilities (±25%), discount rates (5%), mortality following fractures (±25%), anti-fracture efficacy (±25%), and the price of ABL (±20%). Additionally, a less conservative assumption was tested for hip fracture risk reduction with ABL, using an RR of 0.42 instead of 0.63 based on the effect on major osteoporotic fractures. Additional sensitivity analyses assumed no excess mortality following NHNV fractures and applied Medicare costs to all women, including those aged 50-64. We also examined a ±50% variation in the cost of REMS. Different probabilities of patients tested by REMS ultimately receiving a medication to evaluate the cost-effectiveness of REMS as screening tool in the broader community were studied.

To assess the effect of the joint uncertainty surrounding the model variables, we also undertook a probabilistic sensitivity analysis. In each of the 200 simulations, random values were selected for nearly all model variables based on the assigned distributions (see Appendix B). The results were summarized using a cost-effectiveness acceptability curve, illustrating the percentage of simulations in which REMS was deemed cost-effective across varying thresholds of decision makers’ willingness to pay per QALY gained.

For all US female population aged 50 and over, the lifetime prospective economic benefits of the REMS/treatment strategy were evaluated. These benefits included fractures prevention, the gain of life years and QALYs, and the savings on fracture costs. Based on clinical expert consensus, REMS was expected to raise the proportion of US women undergoing osteoporosis treatment by 5% in base case, with additional scenarios considering 2.5% and 10% increases based on clinical expert consensus. In an additional scenario analysis, we tested the assumption that REMS could detect only 85% of the osteoporosis population, based on the reported average positive predictive value in the study of Cortet et al.^39^

### Model validation

The robustness of the model was evaluated through multiple efforts, including protocol validation by a US clinical expert, sensitivity analyses with alternative parameters and assumptions—where the direction of changes was confirmed to align with expectations—and a comparison of model-predicted outcomes (such as fracture numbers and life expectancy) with published data. Specifically, the model estimates that approximately 980 000 fractures (including 205 000 hip fractures) occur annually among US women aged 50 and older with osteoporosis. This figure is consistent with data from the Bone Health and Osteoporosis Foundation, which reports around 2 million fractures, including 300 000 at the hip. The discrepancy is due to a significant proportion of fractures occurring in men and women without low bone mass.

## Results

### Base-case analysis

Table 2 presents the incremental lifetime costs, number of fractures, QALYs, and the ICER (expressed in US dollars per QALY gained) of REMS followed by treatment compared to no REMS/treatment in US women aged 50 yr and above. Under conditions of full medication adherence, the incremental lifetime costs per patient were $5038, with healthcare savings of $13 451 offset by treatment costs of $18 489. The REMS/treatment strategy resulted in the prevention of 0.304 fractures per patient and an increase of 0.1486 QALYs, yielding an ICER of $33 891 per QALY gained. This value is far below the US cost-effectiveness threshold of $100 000 per QALY, suggesting the cost-effectiveness of the strategy.

**Table 2:** Incremental lifetime costs, QALYs, fracture number, and ICER of REMS with treatment vs no REMS/treatment in US women aged 50+.

Lifetime per patient	REMS Full adherence	REMS Real-world adherence
**Total costs**	5038	3433
** Healthcare costs**	−13 451	−6579
** Treatment costs**	18 489	10 012
**Number of fractures prevented**	0.304	0.160
**Quality-adjusted life years**	0.1486	0.0698
****ICER** ^**a**^ **of REMS vs no REMS****	33 891	49 198

a Expressed in cost per quality adjusted life years gained.

Abbreviations: ICER, incremental cost-effectiveness ratio; QALY, quality-adjusted life year; REMS, radiofrequency echographic multi-spectrometry.

In the scenario reflecting real-world medication adherence, the incremental lifetime costs are reduced to $3433 per patient, with healthcare savings of $6579. This adherence level leads to the prevention of 0.160 fractures per patient and an increase of 0.0698 QALYs. The ICER was estimated at $49198per QALY gained, which remains below the US cost-effectiveness thresholds.

### Sensitivity analyses

**Figure 1:**
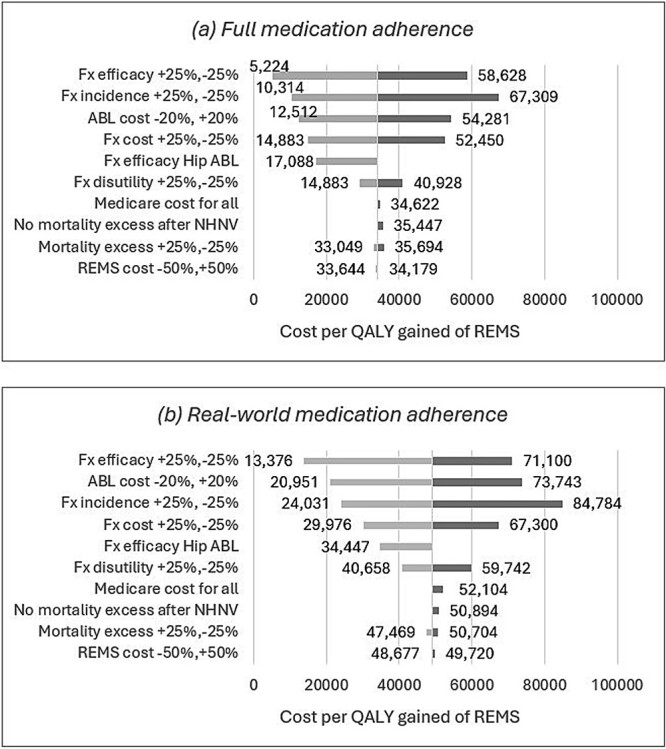
Tornado diagrams: cost-effectiveness of REMS followed by treatment vs no REMS/treatment with (A) full medication adherence and (B) real-world medication adherence. Abbreviations: ABL, abaloparatide; FX, fracture; NHNV, non-hip non-vertebral; QALY, quality-adjusted life-years; REMS, radiofrequency echographic multi-spectrometry.

**Figure 2:**
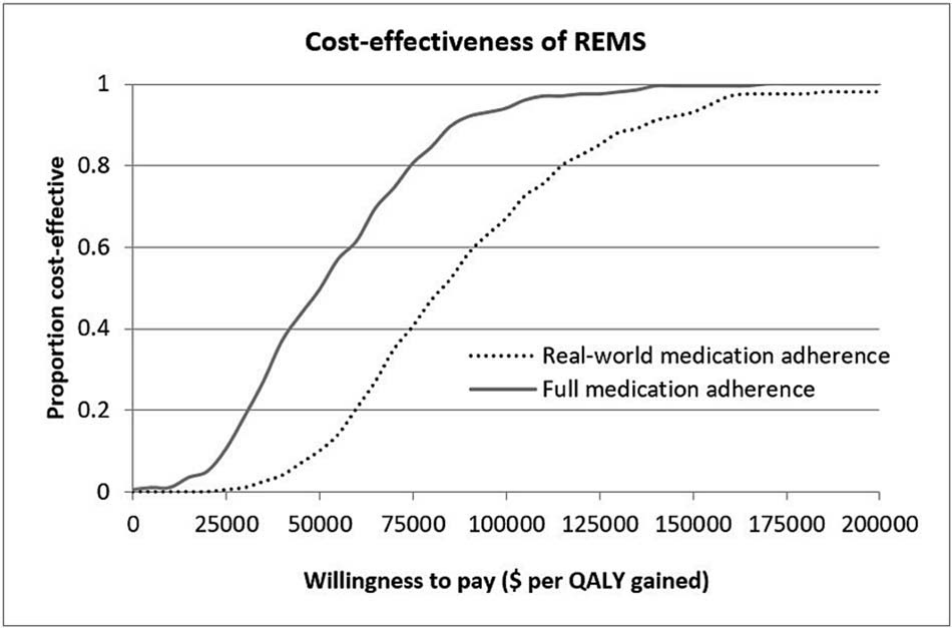
Cost-effectiveness acceptability curves of REMS followed by treatment vs no REMS/treatment in US women with full and real-world medication adherence. The curves illustrate the probability that the REMS/treatment strategy is cost-effective at various cost-per-QALY gained thresholds. This is represented by the proportion of simulations where the REMS/treatment strategy proves to be cost-effective compared to no REMS. A strategy with a probability exceeding 50% is considered the cost-effective choice. It should be noted that due to the random selection of variables in the probabilistic sensitivity analyses, some simulations might depict unrealistic scenarios. For example, these could include instances of low incidence combined with low treatment efficacy and/or low fracture costs. Abbreviations: QALY, quality-adjusted life-years; REMS, radiofrequency echographic multi-spectrometry.

The sensitivity analyses unequivocally affirmed the cost-effectiveness of the REMS/treatment strategy. Across all 1-way sensitivity analyses, regardless of adherence level, the ICER of REMS followed by treatment consistently fell below the US cost-effectiveness thresholds (Figure 1). The tornado diagrams unveiled that fracture incidence, fracture costs, drug treatment efficacy, and the cost of ABL markedly influenced the ICER. Expected increased fracture incidence and costs would thus improve the cost-effectiveness in the future. On the other hand, excess mortality following fractures (including the absence of excess mortality after NHNV fractures) and the use of Medicare costs for the entire population had only a marginal impact on the ICER. Notably, employing a higher efficacy for ABL in hip fracture nearly halved the ICER, revealing that the use of very effective drugs improves the ICER of REMS. Conversely, the impact of REMS cost on the ICER was marginal, registering a mere 1.5% variation for a 50% alteration in costs. Assuming treatment for only 10% of REMS patients resulted in a moderate increase in the ICER. Appendix C Table S1 presents additional probabilities of patients who underwent REMS and ultimately received treatment. The ICER even falls below $100 000 when assuming that only 1 out of 20 tested patients would start medication under real-world adherence conditions. Figure 2 illustrates the cost-effectiveness acceptability curves of REMS followed by treatment. The REMS/treatment strategy is the most cost-effective strategy at the US cost-effectiveness threshold of $100 000 per QALY gained, with probabilities of being cost-effective of 66% and 94%, considering real-world and full medication adherence, respectively. Similarly, probabilities of 93% and 99% were estimated at a $150 000 per QALY gained threshold.

### Potential economic benefits of REMS

Enhancing diagnosis and treatment through REMS for 5% of at-risk US women aged 50 yr and above yielded significant health and economic benefits, as detailed in Table 3. With real-world medication adherence, REMS resulted in about 43 500 discounted QALYs gained and 31 000 undiscounted life years saved. Full medication adherence increased these benefits to 93 000 QALYs and 67 000 life years saved. The strategy also prevented about 100 000 fractures under real world adherence and 190 000 fractures under full medication adherence. Economically, the REMS/treatment strategy over lifetime saved $4.1 billion in fracture costs with real-world adherence and $8.4 billion with full medication adherence. The additional treatment costs over 6.5 yr maximum were estimated at 11.5 and 6.2 billion in case of full and real-world medication adherence, respectively. Assuming a yearly societal economic burden of fractures of 55 billion in the United States,^3^ the extra additional treatment costs would thus represent 3.2% and 1.7% of societal fractures costs under full and real-world medication adherence, respectively. Additional scenarios with 2.5% and 10% increases in patient numbers and those with the assumption that REMS could detect only 85% of the osteoporosis population are included in Appendix C Table S2.

**Table 3:** Potential economic benefits of REMS through enhanced diagnosis and treatment for 5% of at-risk US women aged 50 yr and above.

	REMS Full adherence	REMS Real-world adherence
**Quality adjusted life years gained (discounted)**	92 753	43 536
**Life years saved (undiscounted)**	67 144	30 823
**Fractures prevented**	189 205	100 265
**Fractures costs saved (US$)**	8.4 billion	4.1 billion
**Additional treatment costs (US$)^a^**	11.5 billion (+3.2%)	6.2 billion (+1.7%)

a In parentheses, the extra additional treatment costs compared to the yearly societal economic burden of fractures in the United States are presented.

Abbreviation: REMS, radiofrequency echographic multi-spectrometry.

## Discussion

This study suggests that REMS followed by treatment is associated with improved health outcomes, including more QALYs, fewer fractures, and reduced fracture-related costs compared to no diagnosis and treatment. The ICER of REMS was estimated at $33 891 and $49 198 per QALY gained under full adherence and real-world adherence scenarios, respectively. These values are below the US cost-effectiveness threshold of $100 000 per QALY,^38^ supporting the cost-effectiveness of REMS. Sensitivity analyses revealed that the cost-effectiveness of REMS improved with higher fracture incidence, increased fracture costs, or improved treatment efficacy, which would be expected in the future thereby reinforcing the cost-effectiveness of REMS moving forward. Improved medication adherence also positively impacted cost-effectiveness, although REMS was already cost-effective under real-world adherence conditions. In the hypothetical scenario assuming 100% effectiveness of the medications, REMS/treatment strategy would result in dominance, leading to significant cost-savings. On the other hand, the costs of REMS had only a very minor effect on the cost-effectiveness of the strategy. Notably, REMS would be cost-effective in routine clinical practice even if only a small proportion of tested patients are treated, already achieving cost-effectiveness if just 1 in 20 patients receives treatment. Our analysis also investigated the potential economic benefits of using REMS in clinical practice. Despite uncertainty in the expected population reach, the potential benefits may be significant, including the prevention of at least 100 000 fractures and 43 000 QALYs over a lifetime under real-world medication adherence, assuming that 5% of women at HR and VHR of fractures are diagnosed and treated, and that REMS effectively detects all patients with osteoporosis. These estimates indicate the substantial potential benefits of broad REMS implementation.

This study is the first comprehensive full economic evaluation of REMS, making direct comparisons to existing literature difficult. A recent Italian study^10^ found that REMS is associated with lower direct healthcare costs compared to DXA, although health outcomes were not assessed. In our study, we conservatively assumed the cost of REMS to be similar to DXA. Growing literature demonstrates a strong correlation between DXA and REMS measures,^7^^,^^8^^,^^40^ supporting REMS as a viable tool for osteoporosis management. As cost-effectiveness becomes increasingly crucial for policymakers, it is garnering more attention in osteoporosis research.^41^ Our study uniquely evaluated the economic value of REMS across the entire US female population aged over 50, stratified by HR and VHR. Unlike previous studies that have typically focused on the cost-effectiveness of sequential or monotherapy treatments within specific high-risk populations,^13^^,^^24^^,^^42^ our study’s novel approach encompasses both HR and VHRgroups, providing a comprehensive analysis of REMS’s economic impact on a broader demographic.

The results of this economic study should be interpreted considering several limitations. Many of these limitations are consistent with those reported in the recent economic analysis of sequential treatment with ABL in the United States,^13^^,^^14^ including assumptions about hip fracture efficacy of ALN, medication adherence data, and other model inputs and assumptions. Specific to this REMS cost-effectiveness study, one limitation is the uncertain impact of REMS on medication adherence. Real-world studies, determining the optimal follow-up period between successive REMS scans, would be beneficial. The assumption that REMS costs in the United States are similar to DXA costs is another potential limitation. In contrast to DXA, an X-ray certified technician is not required for REMS, resulting in lower associated fees. Additionally, REMS devices require less space than DXA machines, contributing to further cost savings. Future adjustments may thus be necessary to reflect actual costs accurately, although it is reasonable to expect that REMS cost will remain lower than that of DXA. However, the reported sensitivity analysis showed that REMS cost had a definitely negligible impact on ICER. Furthermore, the classification of patients as HR and VHR varies between countries. Criteria beyond a recent fracture and a BMD T-score ≤ −2.5 could influence VHR classification, suggesting that a more comprehensive definition might be required. Our analysis was restricted to women aged 50 yr and older, but REMS could also be beneficial for men and younger women. Additionally, the non-ionizing radiation emitted by REMS makes it suitable for use in previously underserved populations, including pregnant women, women of childbearing age, children, and frail patients, whether at the bedside, in primary care, low-resource settings, or even at home.^9^ Furthermore, REMS may also be more sensitive than DXA for some conditions like type 2 diabetes.^43^ The REMS technique also offers the advantage of not only measuring bone density but also generating a fragility score, providing a comprehensive assessment of both bone quantity and quality and 5-yr fracture risk. Our analysis however specifically concentrated on the cost-effectiveness of REMS followed by treatment, in comparison to no diagnosis, without directly assessing its economic value relative to DXA. On the other hand, while available published data suggest that REMS can predict incident fractures comparably to DXA, further research is needed to confirm these findings across larger populations as long as REMS data become routinely available similarly to DXA ones.

Finally, transferability of our findings to other settings and countries may be uncertain due to differences in fracture incidence, drug costs, and fracture-related costs, although it is likely that REMS will provide similar economic outcomes in different contexts.

In conclusion, this study suggests that REMS followed by treatment is a cost-effective strategy for the diagnosis and management of osteoporosis in US women aged 50 yr and over, offering substantial potential economic benefits and improved health outcomes.

## Supplementary Material

Appendices_ziae138

## Data Availability

Data may be available from the corresponding author upon reasonable request for academic studies.
