# Supplementary material for: Cost-effectiveness of radiofrequency echographic multi-spectrometry for the diagnosis of osteoporosis in the United States
Source: JBMR Plus. 2024 Nov 6;9(1):ziae138. doi: 10.1093/jbmrpl/ziae138 (PMC11631096; doi:10.1093/jbmrpl/ziae138)
Supplement: Appendices_ziae138 [file appendices_ziae138.docx]

# Appendix A

# Table 1. CHEERS 2022 Checklist

| **Topic** | **No.** | **Item** | **Location where item is reported** |
| --- | --- | --- | --- |
| **Title** |  |  |  |
|  | 1 | Identify the study as an economic evaluation and specify the interventions being compared. | Title |
| **Abstract** |  |  |  |
|  | 2 | Provide a structured summary that highlights context, key methods, results, and alternative analyses. | Abstract |
| **Introduction** |  |  |  |
| **Background and objectives** | 3 | Give the context for the study, the study question, and its practical relevance for decision making in policy or practice. | Introduction |
| **Methods** |  |  |  |
| **Health economic analysis plan** | 4 | Indicate whether a health economic analysis plan was developed and where available. | Methods |
| **Study population** | 5 | Describe characteristics of the study population (such as age range, demographics, socioeconomic, or clinical characteristics). | Methods |
| **Setting and location** | 6 | Provide relevant contextual information that may influence findings. | Methods |
| **Comparators** | 7 | Describe the interventions or strategies being compared and why chosen. | Methods |
| **Perspective** | 8 | State the perspective(s) adopted by the study and why chosen. | Methods |
| **Time horizon** | 9 | State the time horizon for the study and why appropriate. | Methods |
| **Discount rate** | 10 | Report the discount rate(s) and reason chosen. | Methods |
| **Selection of outcomes** | 11 | Describe what outcomes were used as the measure(s) of benefit(s) and harm(s). | Methods |
| **Measurement of outcomes** | 12 | Describe how outcomes used to capture benefit(s) and harm(s) were measured. | Methods |
| **Valuation of outcomes** | 13 | Describe the population and methods used to measure and value outcomes. | Methods |
| **Measurement and valuation of resources and costs** | 14 | Describe how costs were valued. | Methods |
| **Currency, price date, and conversion** | 15 | Report the dates of the estimated resource quantities and unit costs, plus the currency and year of conversion. | Methods |
| **Rationale and description of model** | 16 | If modelling is used, describe in detail, and why used. Report if the model is publicly available and where it can be accessed. | Methods |
| **Analytics and assumptions** | 17 | Describe any methods for analysing or statistically transforming data, any extrapolation methods, and approaches for validating any model used. | Methods |
| **Characterising heterogeneity** | 18 | Describe any methods used for estimating how the results of the study vary for subgroups. | Methods |
| **Characterising distributional effects** | 19 | Describe how impacts are distributed across different individuals or adjustments made to reflect priority populations. | Methods |
| **Characterising uncertainty** | 20 | Describe methods to characterise any sources of uncertainty in the analysis. | Methods |
| **Approach to engagement with patients and others affected by the study** | 21 | Describe any approaches to engage patients or service recipients, the public, communities, or stakeholders (such as clinicians or payers) in the design of the study. | methods |
| **Results** |  |  |  |
| **Study parameters** | 22 | Report all analytic inputs (such as values, ranges, references) including uncertainty or distributional assumptions. | Results |
| **Summary of main results** | 23 | Report the mean values for the main categories of costs and outcomes of interest and summarise them in the most appropriate overall measure. | Results |
| **Effect of uncertainty** | 24 | Describe how uncertainty about analytic judgments, inputs, or projections affect findings. Report the effect of choice of discount rate and time horizon, if applicable. | Results |
| **Effect of engagement with patients and others affected by the study** | 25 | Report on any difference patient/service recipient, public, community, or stakeholder involvement made to the approach or findings of the study | NR |
| **Discussion** |  |  |  |
| **Study findings, limitations, generalisability, and current knowledge** | 26 | Report key findings, limitations, ethical or equity considerations not captured, and how these could affect patients, policy, or practice. | Discussion |
| **Other relevant information** |  |  |  |
| **Source of funding** | 27 | Describe how the study was funded and any role of the funder in the identification, design, conduct, and reporting of the analysis | Funding |
| **Conflicts of interest** | 28 | Report authors conflicts of interest according to journal or International Committee of Medical Journal Editors requirements. | Conflict of interest |

*From:* Husereau D, Drummond M, Augustovski F, et al. Consolidated Health Economic Evaluation Reporting Standards 2022 (CHEERS 2022) Explanation and Elaboration: A Report of the ISPOR CHEERS II Good Practices Task Force. Value Health 2022;25.

# Table 2. IOF-ESCEO Osteoporosis-specific checklist

| Item | Item no. | Recommendation | Reported on page no. / line no. |
| --- | --- | --- | --- |
| Transition probabilities | 1 | Report the transition probabilities and how they were estimated (including increased fracture risk) | Methods |
| Excess mortality after fractures | 2 | Describe approaches and data sources used for the excess mortality after fractures | Methods |
| Fractures costs | 3 | Describe approaches and data sources used for fractures costs | Methods |
| Fractures effects on utility | 4 | Describe approaches and data sources used for the effects of fractures on utility | Methods |
| Treatment effect during treatment | 5 | Describe fully the methods used for the identiﬁcation, selection and synthesis of clinical effectiveness data (per fracture site) | Methods |
| Treatment effect after discontinuation | 6 | Describe fully the methods used for the treatment effect after discontinuation | Methods |
| Medication adherence | 7 | Describe approaches and data sources used for modelling medication adherence | Methods |
| Treatment costs | 8 | Describe approaches and data sources used for therapy costs | Methods |
| Treatment side effects | 9 | Describe approaches and data sources used for costs and utilities effects of adverse events | Methods |

# Table 3. IOF-ESCEO checklist for the design and conduct of an economic evaluation in osteoporosis

| Type of economic evaluation |  |
| --- | --- |
| - Cost-utility analysis using QALY as outcome | YES |
| Method for the conduct of economic evaluation |  |
| - A model based economic evaluation | YES |
| Modelling technique |  |
| - Lifetime horizon | YES |
| - Markov model is appropriate (6 months/1 year cycle length) | YES (6 months) |
| - Avoid hierarchy of fractures and restrictions after fracture events | YES |
| - Hip, clinical vertebral and non-vertebral non-hip fracture | YES |
| Base-case analysis and population |  |
| - Multiple scenarios: age range, BMD and fracture risk scenarios | YES |
| - At least a scenario including a 10-year risk of a major osteoporotic fracture equal to 20% or with a BMD T-score ≤-2.5 with or without fractures | YES |
| - The FRAX® or GARVAN® tools can be used to model fracture risk | NA |
| - Increased risk after fracture events within the model | YES |
| Mortality   - An additional effect (on costs and/or utility) after multiple fractures | YES |
| - Excess mortality after hip fractures | YES |
| - Proportion attribute to the fracture (e.g. 25-30%) | YES |
| Fracture costs and utility |  |
| - Societal and/or healthcare payor perspective | YES |
| Acute fracture costs |  |
| - Long-term costs after hip fracture (attributable to the fracture) | YES |
| - First year and subsequent years effects of fractures on disutility | YES |
| - National ICUROS data if available | YES (whole ICUROS study) |
| Treatment characteristics |  |
| - Treatment duration similar to guidelines or RCTs | YES |
| - Comparators: no treatment and relevant active osteoporotic agent(s) | YES |
| - Sequential therapy may be considered as intervention/comparators | YES |
| - Efficacy data from RCTs, (network) meta-analysis | YES |
| - In the absence of hip/wrist specific efficacy data, use of non-vertebral or clinical fracture efficacy data | YES |
| - Treatment effects after discontinuation depending on treatment | YES |
| - Medication adherence as sensitivity analysis | YES (base case) |
| - Drug costs and administration/monitoring costs | YES |
| Adverse events | YES |

# Appendix B

# Table 1. Distributions for the probabilistic sensitivity analyses

| Parameter | | | Distribution |  |  |
| --- | --- | --- | --- | --- | --- |
| Fracture risk (at age 70-74) | | | *Beta* |  |  |
| Hip | | | Alpha = 14,578, Beta = 3,700,000 |  |  |
| Vertebral | | | Alpha = 17,501, Beta = 3,700,000 |  |  |
| NHNV | | | Alpha = 77,922, Beta = 3,700,000 |  |  |
| Increased relative risk of fractures due to osteoporosis (at age 70-79) | | | *Log normal* |  |  |
| Hip | | | Mean = 0.813, SD = 0.033 |  |  |
| Vertebral | | | Mean = 0.572, SD = 0.186 |  |  |
| NHNV | | | Mean = 0.476, SD = 0.055 |  |  |
| Treatment relative risk reduction | | *Log normal* | |  |  |
| ABL – hip | | | Mean = -0.562, SD = 0.291 |  |  |
| ABL – vertebral | | Mean = -1.966, SD = 0.517 | |  |  |
| ABL – NHNV | | Mean = -1.204, SD = 0.358 | |  |  |
| ALN – hip | | Mean = -0.478, SD = 0.229 | |  |  |
| ALN – vertebral | | Mean = -0.580, SD = 0.100 | |  |  |
| ALN – NHNV | | Mean = -0.211, SD = 0.091 | |  |  |
| Excess mortality after fractures | | | *Log normal* |  |  |
| Hip, vertebral, 0-6 months | | | Mean = 1.512, SD = 0.128 |  |  |
| Hip, vertebral, 7-12 months | | | Mean = 0.562, SD = 0.106 |  |  |
| Hip, vertebral, subsequent years | | | Mean = 0.576, SD = 0.150 |  |  |
| Other, 1^st^ year | | | Mean = 0.322, SD = 0.081 |  |  |
| Effects of fracture on utilities | | | *Beta* |  |  |
| Hip, 1^st^ year | | | Alpha = 1307, Beta = 1070 |  |  |
| Hip, subsequent years | | | Alpha = 636, Beta = 104 |  |  |
| Vertebral, 1^st^ year | | | Alpha = 909, Beta = 428 |  |  |
| Vertebral, subsequent years | | | Alpha = 666, Beta = 118 |  |  |
| NHNV, 1^st^ year | | | Alpha = 26, Beta = 7 |  |  |
| NHNV, subsequent years | | | Alpha = 9, Beta = 0.47 |  |  |
| Fracture costs | | |  |  |  |
| Cost of a fracture | | | *Normal (SD=20% of the mean)* |  |  |
| Hip, 1^st^ year | | Mean = 119,613, SD = 23,923 (50-64 y), Mean = 75,658, SD = 15,132 (65+ y) | | | |
| Vertebral, 1^st^ year | | Mean = 60,459, SD = 12,092 (50-64 y), Mean = 35,006, SD = 7001, (65+ y) | | | |
| NHNV, 1^st^ year | | Mean = 29,013, SD = 5,803 (50-64 y), Mean = 31,764, SD = 6353 (65+ y) | | | |
| Hip, subsequent years | | Commercial: Mean = 10,804, SD = 2,161 (year 2), Mean = 7550, SD = 1510 (year 3), Mean = 5947, SD = 1189 (year 4), Mean = 3555, SD = 11 (year 5+)  Medicare: Mean = 7654, SD = 1531 (year 2), mean = 5688, SD = 1138 (year 3), mean = 4052, SD = 810 (year 4), mean = 2898, SD = 580 (year 5+) | | | |
| Vertebral, subsequent years | | Commercial: Mean = 8196, SD = 1639 (year 2), Mean = 4528, SD = 906 (year 3), mean = 2566, SD = 513 (year 4), mean = 1,746, SD = 349 (year 5)  Medicare: Mean = 5760, SD = 1152 (year 2), Mean = 4094, SD = 819 (year 3), Mean = 2982, SD = 586 (year 4), Mean = 2,170, SD = 434 (year 5) | | | |
| NHNV, subsequent years | | Commercial: Mean = 1757, SD = 351 (year 2), Mean = 1097, SD = 219 (year 3), Mean = 642, SD = 128 (year 4), Mean = 377, SD = 75 (year 5)  Medicare: Mean = 2340, SD = 468 (year 2), Mean = 2025, SD = 405 (year 3), Mean = 1335, SD = 267 (year 4), mean = 1263, SD = 253 (year 5) | | | |

*NHNV non-hip non-vertebral, SD standard deviation*

# Appendix C

# Table 1. Cost-effectiveness of REMS followed by treatment (expressed in cost per QALY gained) based on the probability of REMS diagnosing osteoporosis

|  | Full medication adherence | Real-world medication adherence |
| --- | --- | --- |
| 100% of osteoporosis patients diagnosed | 33,891 | 49,198 |
| 50% of osteoporosis patients diagnosed | 34,381 | 50,241 |
| 25% of osteoporosis patients diagnosed | 35,360 | 52,327 |
| 10% of osteoporosis patients diagnosed | 38,296 | 58,584 |
| 5% of osteoporosis patients diagnosed | 43,191 | 69,013 |

**Table 2:** Scenario analyses on the annual potential economic benefits of REMS through enhanced diagnosis and treatment for (A) 2.5% and (B) 10% of at-risk US women aged 50 years and above, and (C) assuming that REMS could detect only 85 % of the osteoporosis population combined with 5% of at-risk US women aged 50 years and above

|  | Full medication adherence | Real-world medication adherence |
| --- | --- | --- |
| ***2.5% increase*** |  |  |
| Quality adjusted life years gained (discounted) | 46,377 | 21,768 |
| Life years saved (undiscounted) | 33,572 | 15,411 |
| Fractures prevented | 94,602 | 50,132 |
| Fractures costs saved | 4.2 billion | 2.1 billion |
| Additional drug costs | 5.8 billion | 3.1 billion |
| Cost per QALY gained | 33,891 | 49,198 |
| ***10% increase*** |  |  |
| Quality adjusted life years gained (discounted) | 185,506 | 87,071 |
| Life years saved (undiscounted) | 134,288 | 61,645 |
| Fractures prevented | 378,410 | 200,530 |
| Fractures costs saved | 16.8 billion | 8.2 billion |
| Additional drug costs | 23.1 billion | 12.5 billion |
| Cost per QALY gained | 33,891 | 49,198 |
| ***85% of osteoporosis population detected (and 5% increase)*** | | |
| Quality adjusted life years gained (discounted) | 78,840 | 37,005 |
| Life years saved (undiscounted) | 57,072 | 26,199 |
| Fractures prevented | 160,824 | 85,225 |
| Fractures costs saved | 7.2 billion | 3.5 billion |
| Additional drug costs | 9.9 billion | 5.3 billion |
| Cost per QALY gained | 34,139 | 49,385 |
